# Interaction of tumor cells with the microenvironment

**DOI:** 10.1186/1478-811X-9-18

**Published:** 2011-09-13

**Authors:** Hendrik Ungefroren, Susanne Sebens, Daniel Seidl, Hendrik Lehnert, Ralf Hass

**Affiliations:** 1 First Department of Medicine, University Hospital Schleswig-Holstein, Campus Lübeck, Ratzeburger Allee 160, 23538 Lübeck, Germany; 2 Institute for Experimental Medicine c/o Department of Internal Medicine I, University Hospital Schleswig-Holstein, Campus Kiel, Arnold-Heller-Strasse 3, Haus 6, 24105 Kiel, Germany; 3 Department of Radiation Oncology, University Hospital Schleswig-Holstein, Campus Lübeck, Ratzeburger Allee 160, 23538 Lübeck, Germany; 4 Laboratory of Biochemistry and Tumor Biology, Department of Obstetrics and Gynecology, Medical University, Hannover, Carl-Neuberg-Strasse 1, 30625 Hannover, Germany

**Keywords:** cancer-associated fibroblast, cell migration, epithelial-to mesenchymal transition, extracellular matrix, mammary adenocarcinoma, pancreatic ductal adenocarcinoma, tumor-associated macrophage, tumor stroma, metastasis

## Abstract

Recent advances in tumor biology have revealed that a detailed analysis of the complex interactions of tumor cells with their adjacent microenvironment (tumor stroma) is mandatory in order to understand the various mechanisms involved in tumor growth and the development of metastasis. The mutual interactions between tumor cells and cellular and non-cellular components (extracellular matrix = ECM) of the tumor microenvironment will eventually lead to a loss of tissue homeostasis and promote tumor development and progression. Thus, interactions of genetically altered tumor cells and the ECM on the one hand and reactive non-neoplastic cells on the other hand essentially control most aspects of tumorigenesis such as epithelial-mesenchymal-transition (EMT), migration, invasion (*i.e. *migration through connective tissue), metastasis formation, neovascularisation, apoptosis and chemotherapeutic drug resistance. In this mini-review we will focus on these issues that were recently raised by two review articles in CCS.

## Introduction

The complex process of metastasis formation can be divided into several stages: emigration from the primary tumor, invasion of the surrounding tissue and its extracellular matrix (ECM), intravasation into the circulation or the lymphatic system via transmigration through the endothelial lining and the basement membrane, and finally extravasation and metastasis formation at target sites. During each stage, tumor cells have to detach, migrate, invade, adapt and re-attach by involving matrix degrading enzymes and mechanical processes such as cell adhesion, changes of cell fate, cell movements and motility, and the generation of forces. Indeed, an understanding of the invasion process is only possible in the context of detailed insights into the cancer cell's interactions with the microenvironment. These interactions are determined by structural and biochemical properties of the ECM as well as by communication with surrounding non-neoplastic cells such as endothelial cells (ECs, during the process of transendothelial migration), cancer-associated fibroblasts (CAFs), mesenchymal stem cells (MSC), and a variety of different immune cells including lymphocytes and tumor-associated macrophages (TAMs). Since these multiple interactions with the tumor stroma determine not only cancer growth and metastasis but may also develop protective effects with respect to the tumor cells' drug sensitivity/resistance, the tumor stroma also has to be considered as a potential therapeutic target. Specifically, a deeper understanding of these interactions will elucidate the mechanisms of action of classical drugs that have been discovered by empirical approaches and, even more appealing, will facilitate the design and development of novel mechanistically-acting or even individually-designed drugs. This particularly applies for tumors exhibiting a pronounced stromal compartment such as invasive mammary adenocarcinoma (MaCa) and the highly malignant pancreatic ductal adenocarcinoma (PDAC), the latter still presenting as largely resistant to current drug-based therapies. In this mini-review, we refer to two articles which recently appeared in this journal [1,2] describing the major types of tumor stroma interactions (cancer cell with non-neoplastic cells and cancer cell with ECM). The issues raised in these articles will be discussed here in a wider context, including the current view on the role of the tumor stroma in metastasis formation. Special attention is devoted to the dialogue of tumor cells with TAMs, CAFs, and ECs and the role of transforming growth factor (TGF)-β in the regulation of cancer cell migration and invasion. We extend the data presented by Brabek *et al*. [1] and Calorini & Bianchini [2] by highlighting those interactions that are already exploited, or are potentially suitable for targeted therapeutic intervention.

### Cancer cell interactions with the ECM

Matrix invasion is a crucial prerequisite for metastasis and has to be regarded largely as a mechanical process dependent on the expression of adhesion molecules and matrix degrading enzymes. As outlined by Brabek et al. [1], the architecture and composition of the microenvironment in terms of structural and biochemical properties of the ECM (fiber network morphology collagen content, fiber thickness, extent of intrafibrillar cross-links, and the ratio mesh size-diameter of the migrating cell) determines the degree of resistance the moving cell encounters. This in turn will determine the migration strategy and efficiency of cancer cell invasion. Tumor cells are capable of mechanosensing the composition of the ECM which is facilitated by integrin-mediated adhesions and downstream mechanosensor proteins such as focal adhesion kinase (p125^FAK^). On the one hand, increased "stiffness" evokes focal adhesions and increases RhoA-mediated actomyosin contraction. Thus, tissue rigidity can potently stimulate directed cell migration [3]. On the other hand, the mechanical properties of the ECM can be remodeled by tumor cells leading to characteristic stiffening of the tumor tissue through collagen crosslinking and increased focal adhesion formation in breast cancer [4]. In addition, contact guidance which is the aligning behavior mediated by mechanosensory integrins also determines the migratory behavior of the tumor cells. Inhibition of integrin signaling represses invasion and hence integrins and their downstream mediators represent viable therapeutic targets for anti-cancer treatment. In fact, targeting of several integrins, particularly β1 integrin [5] is currently evaluated in preclinical or clinical studies in various tumor types including αvβ3 (Vitaxin, MEDI-522), αvβ3 and αvβ5 (Cilengitide, EMD 121974), αv integrins (CNTO 95), α5β1-I and αvβ3 (ATN-161), α2 integrin subunit (E7820) and α5β1 integrin (Volociximab, Eos-200-4, M-200) [reviewed in Ref. [6]]. Reduction of lysyl oxidase (LOX), a copper-dependent amine oxidase that catalyses the crosslinking of collagens, elastin, and fibrillin in the ECM [7] reduces matrix stiffening and thereby impedes malignancy and affects tumor development in MaCa [4]. Furthermore, hydrogen peroxide which is generated as a metabolic product of LOX activity, stimulates activity of the small GTPase Rac1 [8] and thereby enhances the migratory/invasive behavior of tumor cells [9]. However, although LOX appears to represent a promising molecular target [10], LOX inhibitors have not yet been validated in clinical settings.

Cancer cells utilize different strategies for migration, namely collective versus individual movement [11]. During collective movements the tumor cells retain their intracellular junctions [11] while individual migration strategies can be performed either mesenchymal-like or amoeboid. Both strategies are interchangeable with bidirectional transition and differentially controlled by growth factors. Conversion of epithelial cells to individually migrating mesenchymal cells is achieved by a process called epithelial-mesenchymal-transition (EMT). EMT can be induced by several stimuli, *e.g. *TGF-β1 and is regarded as a prerequisite for mesenchymal cancer cell migration and invasion in breast and pancreatic cancer [reviewed in Refs. [12,13]]. This concept has recently raised great attention since besides its role in conveying the ability for individual migration upon tumor cells it also contributes to drug resistance [14], escape from oncogene-induced premature senescence [15], acquisition of stem cell features [16], and resistance to anoikis [17] in various tumors. TGF-β promotes EMT and single cell motility, which enables invasion into blood vessels, while in the absence of TGF-β, cells are restricted to collective movement and lymphatic spread [18]. For mesenchymal invasion, cells adopt a spindle-like shape with pseudopodia, whereas the amoeboid invasion mode is characterized by cycles of expansion and contraction of the cell body and bleb-like protrusions. The amoeboid migration mode has been described in leukocytes and many types of tumor cells which requires little or no receptor-facilitated adhesion to the ECM. Since this process is protease-independent it may be less susceptible to both integrin and matrix metalloprotease (MMP) inhibitors. Indeed, the failure of MMP inhibitors in recent clinical trials to prevent cancer progression points to the possibility that protease-independent mechanisms of invasion may be clinically relevant. For instance, tumor cells may undergo a mesenchymal-to-amoeboid transition after blocking pericellular proteolysis or integrins. Since the spatial organisation of collagen and elastin fibers can determine the mode of invasion, i.e. whether the cells move amoeboid-like, protease-independent, or mesenchymal, it may be appealing to first alter the stiffness of the ECM by treatment with LOX-inhibitors (see above) in order to force cancer cells to adopt a particular mode of invasion and subsequently apply inhibitors that specifically target this invasion mode.

### Cancer cell interactions with non-neoplastic cells

Besides the ECM, non-neoplastic cells in the tumor microenvironment strongly impact on tumor cell migratory and invasive properties. Supporting this idea, the review by Calorini and Biancini [2] critically addresses experimental evidence that macrophages, fibroblasts, ECs, and other types of stromal cells that are not discussed in this article (e.g. MSC) control and alter the tumoral microenvironment by inducing changes facilitating the tumor cells' local and distant dissemination. Moreover, these non-neoplastic cells can change their phenotype upon soluble or physical contact-mediated stimulation by tumor cells towards a tumor-promoting one.

TAMs derived from differentiated monocytes that have been recruited to the reactive stroma in response to tumoral chemotactic factors, or from resident macrophages, represent the major component of the immune infiltrate in MaCa and PDAC [19,20]. There are two major lines connecting macrophages and cancer: i) accumulation of macrophages in tissues of chronic inflammation apparently promotes cancer initiation and progression and ii) a high density of TAMs in tumor tissues often correlates with poor prognosis for cancer patients [21]. Since macrophages are generally important for T cell activation and the initiation of T cell-mediated immune responses, it is not clear whether the opposing effects exerted by TAMs on tumor growth and metastasis development reflect different states of activation acquired by TAMs in the tumor microenvironment or whether multiple subpopulations of TAMs exist within the tumor [22]. Experimental evidence indicates that depending on the stimuli, monocytes can differentiate into pro-inflammatory (M1-) or anti-inflammatory (M2-, alternatively activated) macrophages. TAMs resemble M2 macrophages and are generally thought to promote tumor progression because of their inability to induce T cell activation along with their elevated expression of scavenger and mannose receptors and the release of pro-tumorigenic factors such as TGF-β1, IL-10, pro-angiogenic factors and MMPs [23]. Moreover, elevated levels of IL-10 and TGF-β1 found in the tumoral microenvironment of many tumors such as MaCa and PDAC are believed to mediate a conversion from M1 to M2 macrophages [24].

**Figure 1:**
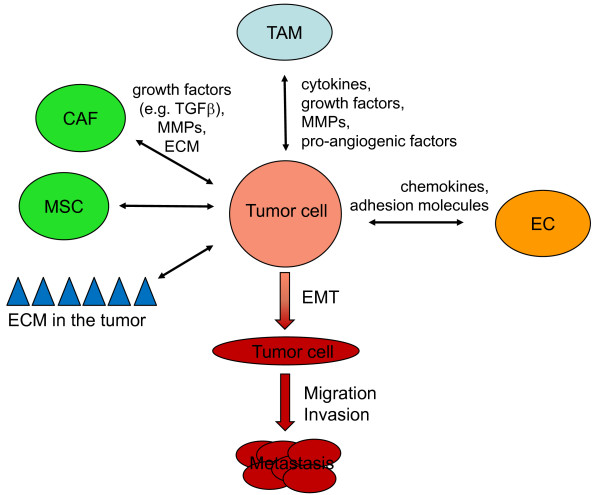
**Reciprocal interactions of tumor cells with the extracellular matrix (ECM), tumor-associated macrophages (TAM), carcinoma-associated fibroblasts (CAF), mesenchymal stem cells (MSC), and endothelial cells (EC)**. These interactions are mediated by direct cell-to-cell contact and/or the release of cytokines, chemokines, growth factors, matrix metalloproteases (MMPs), and ECM proteins. Eventually this results in epithelial-mesenchymal transition (EMT) of tumor cells, their migration, invasion, and dissemination to distant organs and the formation of metastases. For reasons of clarity, other stromal cell populations, *e.g. *lymphocytes and known interactions among different stroma cell populations are not shown.

It is well accepted that TAMs are required for tumor cell migration, invasion, and metastasis formation [25-27]. Altogether, tumor cells exposed to TAMs' prometastatic activity exhibit increased invasiveness and an enhanced capacity to adhere to ECs and thus eventually (and indirectly) facilitate transendothelial migration. Along the same line are observations that tumor cell intravasation (at least in mammary tumors) occurs in association with perivascular macrophages [28]. However, the best characterized pro-tumoral function of TAMs relates to their pro-angiogenic capacities. TAMs generally accumulate in hypoxic areas of the tumor and hypoxia in turn triggers a pro-angiogenic program in these cells. Thereby, TAMs promote the angiogenic switch and neovascularization as well as malignant transition of the tumor cells by secretion of specific pro-angiogenic factors (VEGF, IL-1β, TNF-α, angiogenin, semaphorin 4D), or indirectly through the release of MMP-9. Accordingly, tumor cells co-cultured with macrophages display increased cell migration which is mediated through TNF-α which is released by macrophages [29]. MMPs which are important for ECM degradation and tumor cell invasion through connective tissue can be released by both tumor cells and TAMs. Thus, tumor cells may stimulate TAMs to produce MMPs in a paracrine manner through secretion of interleukins and growth factors. It is also possible that MMPs secreted by TAMs can be recruited to cancer cell membranes and used there by the tumor cells to progress through a specific site. Paracrine stimulation of macrophage-derived MMPs is expected to stimulate protease-dependent modes of cancer cell invasion which are likely susceptible to MMP inhibitors. Another mode of interaction is represented by a GM-CSF/HB-EGF paracrine loop that is utilised by macrophages to promote cancer growth [30] and may be successfully targeted with EGF receptor inhibitors. Indeed, during aging-associated breast cancer development a contribution of signalling events between MMP-7 and HB-EGF has been discussed. Thus, in young normal human mammary epithelial cells (HMEC), MMP-7 can bind to several glycosylation branches of the CD44 receptor isoform variant-3 (CD44v3) which can colocalize with MMP-7 and anchor this proteinase to the cell surface in close vicinity to membrane-bound pro-HB-EGF [31,32]. This reveals a close interaction between MMP-7 and HB-EGF which is not detectable in aging HMEC [32]. Consequently, an extracellular cleavage of proHB-EGF by MMP-7 enhances the availability of soluble HB-EGF (sHB-EGF) which can bind to and interact with the ErbB4 receptor [33,34]. This process can be observed in normal young proliferating HMEC [32]. Conversely, altered expression levels of sHB-EGF and the ErbB4 receptor have been reported in neoplastic breast cancer cells [32,35]. In association with these proliferation signals, previous work has also suggested that MMP-7 can mediate neoplastic growth of mammary epithelial cells via the ErbB4 receptor [34]. The signals relayed by HB-EGF via the ErbB4 receptor involve the mitogen-activated protein kinase (MAPK) pathway and can also be identified in normal and neoplastic breast tissue to mediate an increased proliferation signal [36]. Moreover, adjacent TAMs, CAFs, and MSC within the neoplastic tissue microenvironment contribute to the release of growth factors (Figure 1). Particularly MSC which represent different subpopulations [37] and alter metabolic activities in an hypoxic microenvironment [38] can further enhance the proliferative capacity during maturation and interact with tumor cell populations [39]. In addition, co-culture experiments revealed that senescent human fibroblasts can affect neighboring epithelial cells, for example by increasing the survival and growth of pre-malignant and malignant mammary epithelial cells or by altering the functional differentiation and branching morphogenesis of normal breast epithelial cells [40,41]. Upon appropriate signals from the tumor cells, TAMs also produce and activate other extracellular matrix proteases including the urokinase-type plasminogen activator (uPA) and its receptor, uPAR, that may cause ECM degradation to promote invasion and spreading of tumor cells [42]. The expression of uPAR has been shown to be fairly high in tumor compared to normal, quiescent tissues, which has led to uPAR being proposed as a therapeutic target, as well as a targeting agent, for the treatment of cancer [43].

Another important non-neoplastic cell type found in the tumor stroma is the CAF. CAFs actively contribute to tumor progression [44], like tumor cells are capable of monocyte recruitment, and like TAMs promote neovascularization, cancer cell proliferation, survival, and invasion (Figure 1). In addition, CAFs can be generated as activated fibroblasts or myofibroblasts from normal fibroblasts by stimulation with TGF-β1. Tumor cell-derived TGF-β stimulates reactive oxygen species (ROS)-dependent expression of α-smooth muscle actin in the fibroblasts leading to their differentiation into myofibroblasts [45], offering the theoretical possibility of therapeutically preventing this conversion with ROS scavengers like N-acetyl-cysteine. In this activated state, CAFs produce a variety of cytokines, growth factors and ECM proteins by which they alter both the tumor cells and the stromal microenvironment and promote tumor progression. For instance, CAF-derived hepatocyte growth factor leads to an invasive phenotype and, like TGF-β, can induce EMT in tumor cells. As a consequence, the EMT associated transcription factors Twist1 and 2 may override oncogene-induced premature senescence in cancer cells [16], providing a link between early escape from failsafe programs/prevention of oncogene-induced senescence and the acquisition of invasive features by cancer cells.

By producing ECM proteins, CAFs can determine the biophysical properties of the ECM (thereby indirectly influence cancer cell motility and invasion modes) and act as barrier against tumor-infiltrating immune cells and access of anti-cancer drugs to the tumor cells [46], facilitate cell contacts and motility, and stimulate secreted proteins which in turn stimulate invasiveness, angiogenesis and tissue remodeling. CAF-secreted metalloproteases, such as MMP-3, elicit a Rac1b (a tumor-specific splice variant of Rac1)/COX-2-mediated release of ROS in carcinoma cells which is mandatory for EMT, stemness, and dissemination of metastatic cells [47]. Inhibitors of MMPs, small GTPases, and ROS may thus cross-target CAF-mediated prooncogenic events. Moreover, CAFs from invasive human breast carcinomas promote tumor growth and neoangiogenesis through a SDF-1/CXCR4-dependent recruitment of endothelial progenitor cells [48].

Besides interactions with TAMs and CAFs, the interplay of tumor cells with ECs is also of pivotal importance for tumor progression and the development of metastasis. It is generally assumed that cancer cell migration through connective tissue is too slow and undirected to account for the quick spreading and metastasis formation seen in many tumors, and that cancer cells spread much more quickly and efficiently via lymph or blood vessels to distant sites. The endothelium and the basement membrane constitute a strong physical barrier, hence the process of intravasation is potentially time-consuming and rate-limiting in metastasis development. The mechanisms by which cancer cells can transmigrate through the endothelial lining are not well understood. Signaling cross-talk between cancer cells and ECs may involve upregulation of adhesion molecule expression by the endothelium as well as by the tumor cells, reorganisation of the acto-myosin cytoskeleton, and Src-mediated disruption of endothelial VE-cadherin-β-catenin cell-cell adhesions. Paracellular transmigration through the formation of holes within the monolayer and through induction of EC apoptosis is currently discussed. Similar mechanisms may also operate in the generation of pleural effusions often seen in breast cancer patients or ascites in patients with PDAC [49]. As mentioned above, tumor cell intravasation can occur in association with perivascular macrophages [29] enriched for molecules of the Wnt signaling pathway [50]. Signals from cancer cells (and possibly perivascular macrophages), *e.g. *production of inflammatory cytokines such as TNF-α, and IL-1β promote transmigration and invasion by several mechanisms while signals from ECs like chemokines (GRO-β, IL-8) lead to enhanced contractile force generation and cytoskeletal remodeling. A better understanding of cancer cell transmigration may provide multiple potential targets for therapeutic intervention.

### TGF-β: a growth factor of pivotal importance for cancer cell migration/invasion and metastasis formation

The importance of TGF-β signaling for progression of invasive MaCa and PDAC is well documented [51,52]. Recently, the TGF-β pathway has been identified as one of only 4 signaling pathways with 100% alteration in PDAC [53]. As outlined above, TGF-β can directly regulate cell migration and invasion during later stages of tumor progression by promoting EMT and single cell motility. To induce this pro-migratory function, TGF-β may exploit signaling crosstalk with other oncogenes and small GTPases with known roles in cellular adhesion, migration, and invasion such as Src [54], K-ras [55], and Rac1 [56]. Moreover, TGF-β is involved in many aspects in the dialogue of cancer cells with the non-neoplastic cells of the tumor microenvironment, particularly the generation/conversion of TAMs and CAFs from monocytes and fibroblasts, respectively, and the induction of adhesion molecule expression in neighboring normal cells [57]. However, TGF-β signaling mediated by CAFs can suppress tumor formation and progression in adjacent epithelia [58]. In addition, TGF-β may also directly regulate ECM tension and stiffness (through increasing the expression of ECM proteins by tumor cells or CAFs and/or the function of LOX,) and thereby increasing the oncogenic activities (*e.g. *proliferation) of cancer cells. In mammary carcinoma, TGF-β appears to play a key role in maintaining the mammary epithelial (cancer) stem cell pool, in part by inducing a mesenchymal phenotype, while differentiated, estrogen receptor-positive, luminal cells are unresponsive to TGF-β because the TGFBR2 receptor gene is transcriptionally silent [59].

In human carcinoma, tumor-cell-autonomous expression of TGF-β is often increased, whereas expression of the receptor-dependent signaling components is decreased, mutated, or silenced. TGF-β is an early tumor suppressor that can subsequently promote tumor progression through tumor-cell-autonomous and tumor-stroma interactions resulting in metastasis development, immune evasion, and the stimulation of angiogenesis [58]. This suggests that therapeutic targeting of TGF-β signaling in cancer cells and TAMs is a feasible therapeutic option. TGF-β pathway inhibitors including small and large molecules have now entered clinical trials. Preclinical studies with these inhibitors have shown promise in a variety of different tumor models [60]. More recently, we have discovered that the common Src family kinase inhibitors PP1 and PP2 are powerful inhibitors *in vitro *of TβRI and II [61]. If this also applies to their action *in vivo *they may have the advantage of dually inhibiting oncogenic Src *and *TGF-β signaling and are likely to be much more effective in the treatment of late-stage MaCa and PDAC than agents with single specificity.

## Conclusions

The tumor stroma provides unique structural features that significantly differ from those of the respective normal tissue. On the one hand cancer cells respond to this environment through modulation of cell adhesion/migration molecules (e.g. L1-CAM, integrins), contact guidance, cytoskeletal reorganisation, cell shape changes, as well as secretion of proteolytic enzymes (MMP-7/-9), growth factors (HB-EGF), chemokines, and cytokines (TNFα, TGF-βs). On the other hand, tumor cell-derived signals recruit and activate host cells among which monocytes/TAMs, fibroblasts/CAFs and MSC are the most abundant populations within the tumor microenvironment. Thus, the dynamic and reciprocal interactions between tumor cells and cells of the tumor microenvironment as well as the ECM orchestrate events critical to tumor progression and metastasis formation.

Current efforts are directed towards studying these various types of tumor cell-stromal cell and tumor cell-matrix interactions in diverse experimental settings. Intense investigations in this field have revealed that the mechanisms that allow CAFs and TAMs or the ECM to contribute to tumor progression also determine the sensitivity of tumor cells towards chemotherapeutic drugs. Drug resistance, intrinsic or acquired, essentially limits the efficiency of chemotherapy in many cancer patients and is the result of reduced accessibility or accumulation of a drug in the tumor tissue (*e.g. *as a consequence of increased matrix deposition acting as a physical barrier), but also of protection from induction and execution of apoptosis in cancer cells. Thus, by interacting with the ECM and/or stroma cells, tumor cells become highly protected from apoptosis induction involving alterations in the expression of adhesion molecules such as L1CAM or integrins, or elevated secretion of cytokines (TGF-β) and chemokines. Since many of these molecules and their signaling intermediates are involved in the control of cell migration and invasion, cell survival, and apoptosis at the same time, they represent particularly exciting molecular targets for anti-cancer therapy.

## Abbreviations

CAFs: cancer-associated fibroblasts; ECs: endothelial cells; ECM: extracellular matrix; EMT: epithelial-mesenchymal transition; LOX: lysyl oxidase; MaCa: mammary carcinoma; MSC: mesenchymal stem cell; MMP: matrix metalloprotease; PDAC: pancreatic ductal adenocarcinoma; ROS: reactive oxygen species; TAMs: tumor-associated macrophages; TGF-β: transforming growth factor-beta; uPA: urokinase-type plasminogen activator.

## Competing interests

The authors declare that they have no competing interests.

## Authors' contributions

HU, SS, and RH wrote the manuscript. DS and HL were involved in the conceptualization and discussion of the manuscript. All authors read and approved the final version of the manuscript.
